# Using the metabolite alterations monitoring the AEG-1 expression level and cell biological behaviour of U251 cell in vitro

**DOI:** 10.1371/journal.pone.0291092

**Published:** 2023-09-01

**Authors:** Yurui Sheng, Di Yin, Qingshi Zeng

**Affiliations:** 1 Department of Radiology, The First Affiliated Hospital of Shandong First Medical University & Shandong Provincial Qianfoshan Hospital, Jinan, Shandong, China; 2 Department of Radiology, The Second Hospital of Tianjin Medical University, Tianjin, China; Kyung Hee University, REPUBLIC OF KOREA

## Abstract

Astrocyte elevated gene-1 (AEG-1) is an important oncogene that overexpresses in gliomas and plays a vital role in their occurrence and progression. However, few reports have shown which biomarkers could reflect the level of AEG-1 expression in vivo so far. In recent years, intracellular metabolites monitored by proton magnetic resonance spectroscopy (^1^H MRS) as non-invasive imaging biomarkers have been applied to the precise diagnosis and therapy feedback of gliomas. Therefore, understanding the correlation between ^1^H MRS metabolites and AEG-1 gene expression in U251 cells may help to identify relevant biomarkers. This study constructed three monoclonal AEG-1-knockout U251 cell lines using the clustered regularly interspaced short palindromic repeat (CRISPR) /Cas9 technique and evaluated the biological behaviors and metabolite ratios of these cell lines. With the decline in AEG-1 expression, the apoptosis rate of the AEG-1-knockout cell lines increased. At the same time, the metastatic capacities decreased, and the relative contents of total choline (tCho) and lactate (Lac) were also reduced. In conclusion, deviations in AEG-1 expression influence the apoptosis rate and metastasis capacity of U251 cells, which the ^1^H MRS metabolite ratio could monitor. The tCho/creatinine(Cr) and Lac/Cr ratios positively correlated with the AEG-1 expression and malignant cell behavior. This study may provide potential biomarkers for accurate preoperative diagnosis and future AEG-1-targeting treatment evaluation of gliomas in vivo.

## Introduction

Glioma is a common principal central nervous system (CNS) tumor with high morbidity and mortality, accounting for 15% of all CNS tumors [1, 2]. Several genes drive the development of gliomas, and understanding the changes in tumor genetic molecules can help diagnose, grade, and treat gliomas. With advances in cancer genomics, gene therapy will likely become the mainstay of glioma treatment [3, 4]. Astrocyte elevated gene-1 (AEG-1) is overexpressed in various malignancies, including gliomas, and plays a role in various complex oncogenic signaling cascades intimately involved in tumor development [5]. Relevant studies have confirmed that AEG-1 plays an essential role in all the hallmarks of cancer, including progression, transformation, sustained angiogenesis, evasion of apoptosis, invasion, and metastasis [4, 6, 7]. A related study revealed that AEG-1 knockdown reduced radiation-induced homologous recombination repair activity by inhibiting RFC5 expression, suggesting that AEG-1 may be a reliable radiosensitization target for glioma radiotherapy [8]. Another study showed that silencing AEG-1 promotes temozolomide-induced DNA damage, improving the efficiency of glioma chemotherapy, and reducing immunosuppression due to M2 polarization in glioma cells [9]. Researchers have demonstrated that the downregulation of AEG-1 significantly inhibits the development of vasculogenic mimicry (VM) by regulating VEGF and MMP-2 in glioma cells [10]. Additional studies have demonstrated that AEG-1 is a target of miR542 to promote the proliferation and invasion of glioblastoma [11]. Although the actual mechanism underlying the effect of AEG-1 on glioma remains unclear, AEG-1, as an emerging target, may still potentially contribute to gene-targeted therapy for human glioma. Therefore, it is crucial to search for effective non-invasive examination methods to assess the expression of AEG-1 in glioma cells, which will be conducive to an inchoate diagnosis and complementary therapy.

The rapid and unrestricted proliferation of tumors is an energy- and resource-consuming process, and metabolism is significantly altered during tumor transformation and progression [12]. Warburg et al. observed that various tumor cells exhibit a unique metabolic phenotype in which glucose undergoes the enzymatic production of pyruvate, which remains fermented to lactate even in an aerobic environment, rather than undergoing oxidative phosphorylation. This feature, known as the Warburg effect, is attributed to tissue hypoxia, genetic mutations, and mitochondrial abnormalities in proliferating cancer cells [12]. Altered cellular metabolism is a hallmark of glioma, and the interaction between the tumor genotype and brain microenvironment affects glioma metabolism [12, 13]. Glioma cells exhibit a higher glucose uptake rate than normal cells, which is accompanied by a metabolic switch from oxidative phosphorylation to aerobic glycolysis. Metabolic reprogramming of glioma cells supports excessive cell proliferation and glycolysis, which are usually mediated by the activation of oncogenes or perturbation of tumor suppressor genes. Corresponding oncogenes include isocitrate dehydrogenase (IDH), TP53, epidermal growth factor receptor (EGFR), phosphatase and tensin homolog (PTEN), retinoblastoma gene 1 (rb1), platelet-derived growth factor receptor (PDGFRA), and Neurofibromatin-1(NF1) [14, 15]. Relatively few reports have addressed the effects of AEG-1 on glioma metabolism.

Magnetic resonance spectroscopy (MRS) is a sensitive technique for imaging tissue metabolism and can be used to enhance the specificity of tissue characterization in a non-invasive manner [16, 17]. Hydrogen-proton magnetic resonance spectroscopy (^1^H MRS) is currently widely employed in clinical practice to detect alterations in metabolites in lesions [18–20]. ^1^H MRS provides irreplaceable information for the diagnosis and therapeutic monitoring of tumors, particularly gliomas [21–23]. Studies have shown that the metabolite ratio has a specific value in differentiating treatment-related changes (radiation necrosis and tumor pseudoprogression) from tumor recurrence in gliomas [24–26]. Total choline (tCho) is considered a biomarker reflecting the state of cell membrane turnover and has been widely used to identify tumor areas with excessive cell proliferation and for the therapeutic monitoring of gliomas[27, 28]. The product of the Warburg effect is lactate (Lac), whose level may reflect the metabolic characteristics of gliomas as they develop [29, 30]. However, the acetate (Ace) and succinate (Succ) detections have not been adequately reported in gliomas. ^1^H MRS shows the probability of offering specific biomarkers by measuring metabolic alterations in cancer that precede changes in magnetic resonance image (MRI) appearance [17]. Although ^1^H MRS has been in clinical use for glioma recognition and treatment inspection for some time, the mechanisms of action at the molecular and cellular level of metabolite alterations are poorly understood. Few studies have investigated the relationship between metabolite alterations and oncogenic expression [31]. In in vitro MRS research, the scientific detection of metabolites could be analyzed by combining certain gene expression and cell biological behaviors [23, 25], which could provide excellent feedback on cellular states in tumor progression. These in vitro research achievements may be transformed into in Vivo MRS studies to sufficiently comprehend new clinical MRS discoveries [32].

Recently, clustered, regularly interspaced short palindromic repeat (CRISPR)/Cas9 technology has become a considerably new device for generating RNA-guided nucleases with customizable specificities [33]. Previous studies notarized the feasibility of editing AEG-1 using the CRISPR/Cas9 system in liver cells [34]. The single guide RNA (sgRNA) guides the Cas9 nuclease to the target site through base pairing [35]. Several sgRNAs target specific genes for selection, and disparate sgRNAs build diverse gene-knockout models with different knockout efficiencies.

Accordingly, the present study aimed to generate monoclonal AEG-1-knockout cell lines using three specific sgRNA-CRISPR/Cas9 genome-editing vectors. Next, validate the effect of AEG-1 on biological behavior in the U251 cell line by analyzing cell apoptosis and cycle distribution using flow cytometry and invasion and migration abilities using Transwell and scratch assays, respectively. Furthermore, certain molecular metabolites of the AEG-1 knockout cell lines were measured using ^1^H MRS, the relationship between metabolite changes and AEG-1 expression was explored, and certain metabolite ratios were determined as a noninvasive imaging biomarker for preoperative evaluation and an indicator of targeted therapy for glioma.

## Materials and methods

### Materials

The experimental materials used in the present study are described below.

### Methods

#### Construction of the three multiclonal AEG-1-knockout cell lines

**Fig 1:**
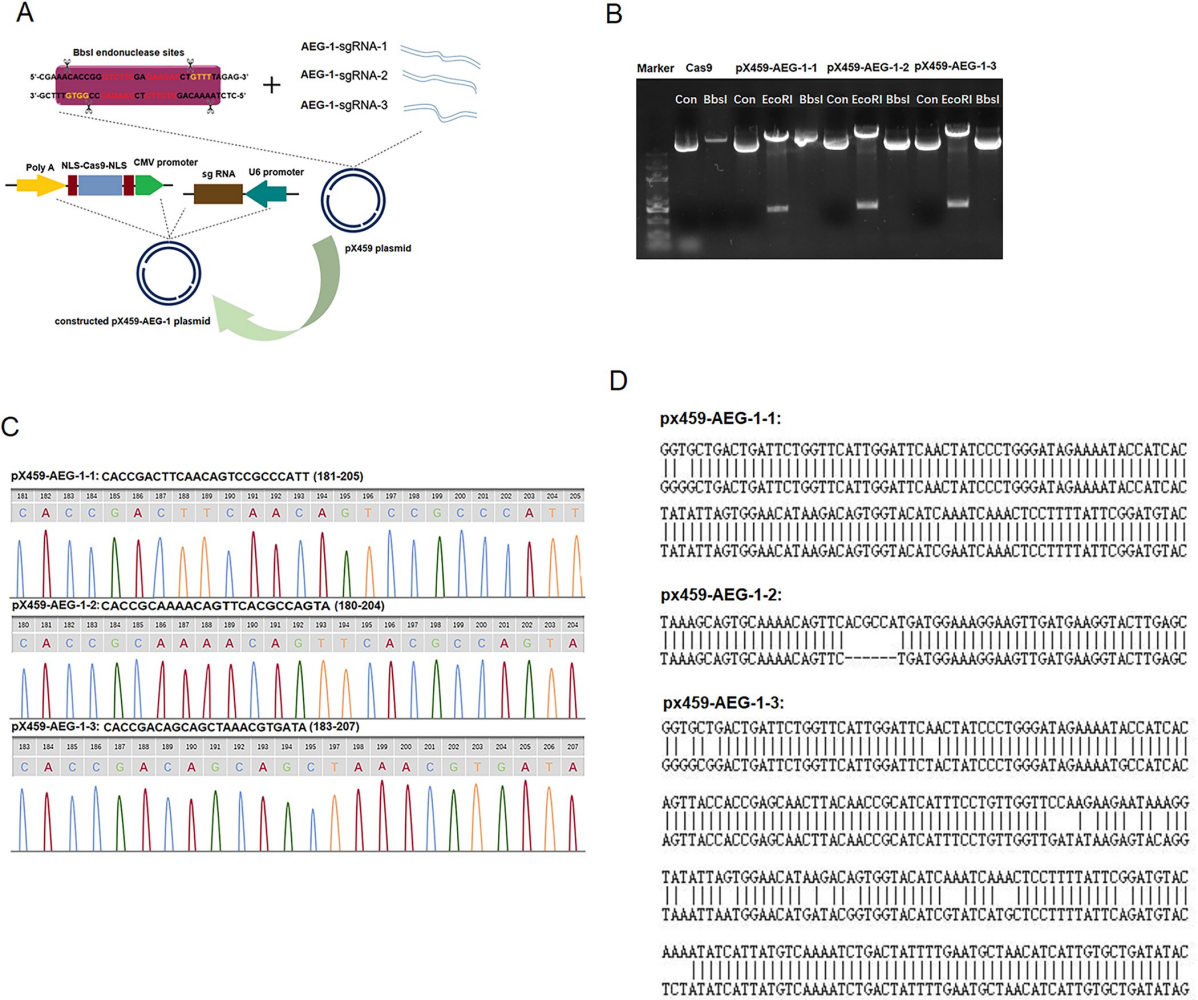
The construction process and results of the three monoclonal AEG-1-knockout cell lines. (A) Schematic diagram of reconstructed pX459 plasmid. (B) Enzyme digestion showed they cannot be cut by Bbs I because the original Bbs I site had a DNA fragment insert. (C) All the plasmids had the donor DNA inserted with the correct open reading frames. (D) TA cloning analysis to identify Indel mutations, three forms of gene mutations were caused.

Three single guide RNA (sgRNA) sequences targeting AEG-1 were designed (Table 1) and scored as the top three on the design website (https://zlab.bio/guide-design-resources). The synthetic sgRNA oligo strand that generates double-stranded DNA was mixed with 1 μL of pX459 plasmid (linearized CRISPR/Cas9 plasmid) and 1 μL of T4 DNA ligase (Fermentas) at 16°C overnight to obtain three pX459-AEG-1 genome-editing vectors (Fig 1A).

**Table 1:** Sequences of the AEG-1 sgRNA oligos.

primer	sequence (5′–3′)
AEG-1-sgRNA-1	Upstream: CACCGACTTCAACAGTCCGCCCATT
	Downstream: AAACAATGGGCGGACTGTTGAAGTC
AEG-1-sgRNA-2	Upstream: CACCGCAAAACAGTTCACGCCATGA
	Downstream: AAACTCATGGCGTGAACTGTTTTGC
AEG-1-sgRNA-3	Upstream: CACCGACAGCAGCGTAAACGTGATA
	Downstream: AAACATTACGTTTACGCTGCTGTC

Next, the three recombinant plasmid DNAs were cut using the restriction enzymes EcoRI (Fermentas) and Bbs I (Fermentas) for preliminary identification and were then subjected to gene sequencing for further identification. U251 cells were cultivated at 90% confluence 24 h before transfection using Lipofectamine 3000 (Invitrogen). A seven-day screening experiment was performed by adding puromycin to the medium (3 μg/mL) to obtain three multiclonal AEG-1-knockout cell lines; each well of each group of cell lines was digested, infinitely diluted, and each cell was individually cultured into a stable monoclonal cell line.

Whole genomes of the monoclonal knockout cell lines were extracted to evaluate the activity of the three reconstructed plasmids at the molecular level. Next, the primers (Table 2) for the corresponding complementary DNAs (cDNAs) were designed. cDNA was cloned into the PMD-18T vector (Takara). Ultimately, the TA-cloning vector for gene sequencing was extracted to estimate the knockout consequences of these sgRNAs. Normal U251 cells were used as a reference, and U251 cells transfected with the empty Cas 9 plasmid served as the direct control group.

**Table 2:** PCR short-chain primers for TA cloning analysis.

Short-chain primer	sequence (5′–3′)	Size of the PCR product
AEG-1-1	Upstream: TCCTGCGTATAAATCTTTG	324
	Downstream: ATCACCCTACCCACCATTT	
AEG-1-2	Upstream: TAAAGCAGTGCAAAACAG	325
	Downstream: ACAGAAACAGAGGACCTT	
AEG-1-3	Upstream: GGTGCTGACTGATTCTGG	293
	Downstream: AAATGGCTTATGGTCGTC	

#### Western blot analysis of the five cell lines

For western blot analysis, 10 μg of protein was separated using 10% SDS-PAGE and transferred to a polyvinyl difluoride membrane. The membrane was steeped in a 5% milk solution for 1 h at 25°C and cultivated at 4°C overnight with rabbit anti-AEG-1 (ab227981; 1:1500; Abcam) and mouse anti-GAPDH (glyceraldehyde-3-phosphate dehydrogenase) (ab8542; 1:5000; Abcam). The primary antibody solution was removed from the membrane. Horseradish peroxidase-labeled goat anti-rabbit antibody (1:4,000; Abcam) and goat anti-mouse antibody (1:2,000; Abcam) were appended separately, and the membrane was incubated at 25°C for 1 h. After washing, the membrane was subjected to development and visualization. Protein expression was quantified using the grayscale ratio in Image J software (Bethesda). The GAPDH expression was used as an internal reference for AEG-1.

#### Flow cytometry analysis of apoptosis and cell cycle distribution

For apoptosis determination, each cell line was cultured with 5 μL of Annexin V and 5 μL of propidium iodide (PI, BD Biosciences) for 15 min at 25°C in the dark and subjected to flow cytometry to measure the apoptosis rate (%). The cell cycle distribution of the cell lines was assessed using flow cytometry. The cell amount was regulated to 2×10^6^ cells/ml and then washed several times, and cells were fastened with 70% ethanol for 12 h at 4°C and cultivated with PI for 30 min at 25°C in the dark. The above steps were repeated thrice. The samples were analyzed by flow cytometry, and the experimental data were analyzed using FlowJo software (Treestar).

#### Transwell invasion assays and scratch wound experiments

A certain amount of Matrigel (Corning) was added to the filter membrane for the Transwell invasion assays. The cells were cultured in DMEM without serum for 24 h. Next, 1×10^6^ cells were engrafted in the respective chambers, and 800 μL of the absolute medium was injected into the inferior chamber. After 24 h of incubation, ten visual fields were randomly picked to count the mean cell. The experimental procedure was replicated thrice with three wells in each group.

Cell migration ability was detected by adopting the ’wound-healing’ assay. The cell lines were inoculated in 6-well plates, and the wounds were fabricated in the contact-suppressed monolayer cell with a germ-free P200 pipette tip, then cultured for 48 h for scratch healing. Adhesive cells were cultured with a new nutrient solution. The condition of wound healing in these groups was assessed, and pictures were taken per group at 0 h and 48 h.

#### Sample preparation for ^1^H NMR spectroscopy and ^1^H NMR spectroscopy

The washed cells were centrifuged for each sample. Then, the cells (approximately 3 × 10^7^ cells) were mingled with hypochlorous acid. Sodium hydroxide was added to adjust the pH to 7.2, and the mixture was centrifuged. The sediment was deserted, and the epipelagic liquid was lyophilized to powder. The lyophilized extracts were placed into a 5 mm MR tube and melted in miscible liquids containing 540 μL pure D_2_O and 60 μL TMSP (2, 2, 3, 3-d (4)-3-(trimethylsilyl) propionic acid sodium salt). TMSP was used as the chemical shift reference. The above steps were repeated thrice in each group.

^1^H NMR spectroscopy was performed using a Bruker Avance 600 MHz 14T spectrometer. A pulse–acquire sequence was employed to perform 128 scans. The TMSP peak appeared at 0.00 ppm and is regarded as the reference signal. The metabolite content was related to the quantified metabolite curve calculus, and we acquired the relative amounts of metabolites through the MestReNova software analysis. The creatinine (Cr)content was used as a reference. The above steps were repeated three times for each group.

#### Statistical analysis

The overall numerical value is presented as the average ± standard deviation. SPSS 23.0 software (Chicago) was used for correlation analysis, and GraphPad Prism 8.0 (La Jolla) was used for diagrams. Pearson’s coefficient of association was used to evaluate pertinence. Two-sided p values < 0.05 were statistically significant for all data analyses.

## Results and discussion

### Results

#### Identification of the pX459-AEG-1 vector and sgRNA activity

As the outcome is shown in Fig 1C, the reconstructed vector could be cut by EcoRI but not by the Bbs I, and the DNA sequencing outcome of all the pX459-AEG-1 vectors is shown in Fig 1C. The two results displayed that synthetic sgRNA oligos were inserted into the pX459 plasmid successfully.

Accurately cloned pX459-AEG-1 vectors were successfully transfected into U251 cells to obtain several monoclonal AEG-1-knockout cell lines. The sequencing results of TA cloning of the AEG-1-knockout cell lines showed (Fig 1D) that they caused three forms of gene mutations. In this study, we will measure the tentative data of five groups of monoclonal cell lines (U251, Cas 9, pX459-AEG-1-1, pX459-AEG-1-2, pX459-AEG-1-3).

#### Expression of AEG-1 and estimation of the knockout efficiency

**Fig 2:**
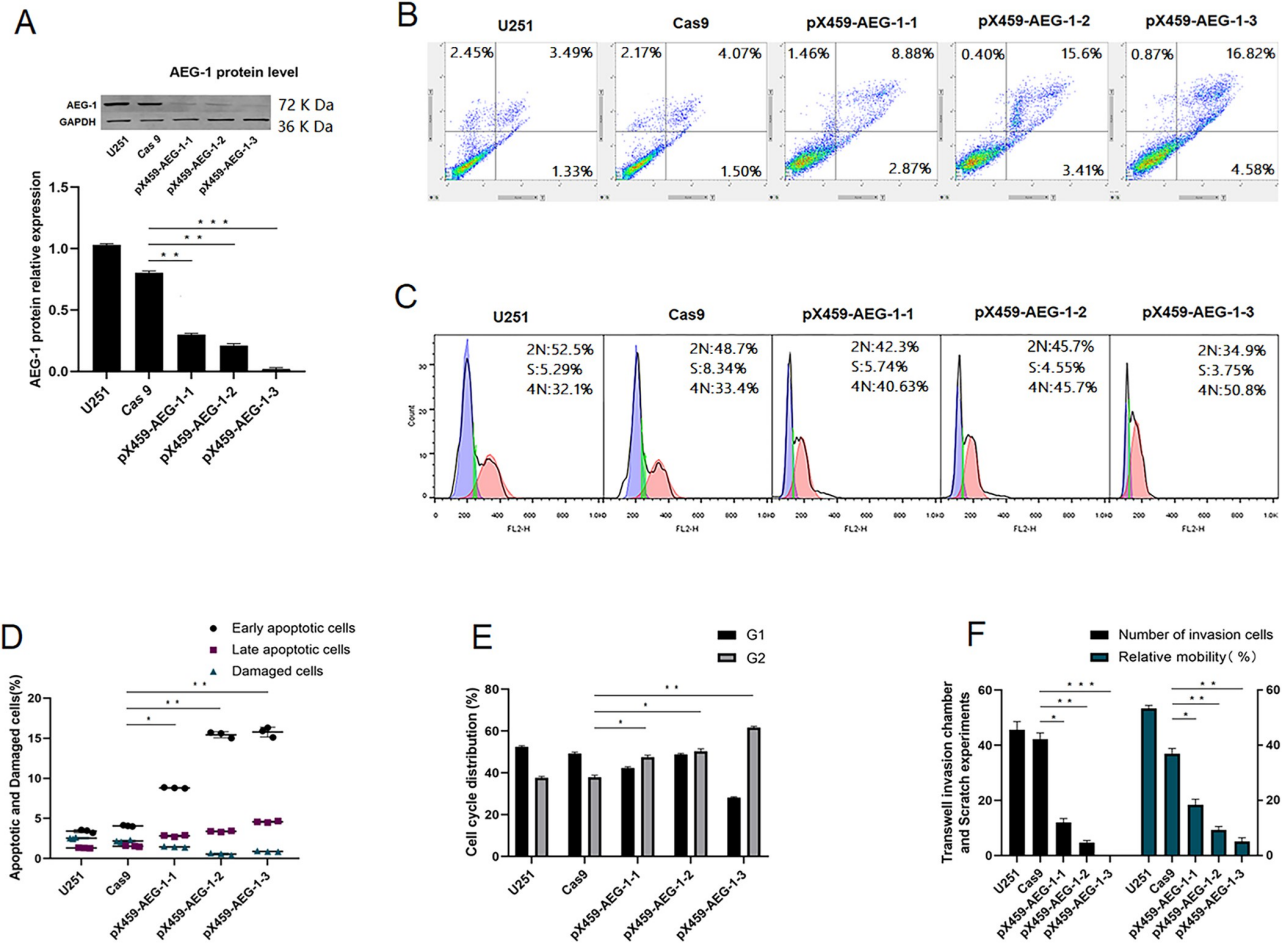
(A) Evaluation of AEG-1 gene expression in the stable AEG-1 gene knockout cell lines. (B)Flow cytometry showed a representative series figure of cell apoptosis. (C)A representative series of cell cycle FACS histograms is shown. (D)The apoptotic rate among the five cell lines. * P<0.05, ** P<0.01, vs control (Cas9) group. (E) The percentage of cells at the G1 and G2 stages of the cell cycle is shown for the five cell lines. * P<0.05, ** P<0.01, vs control (Cas9) group. (F)The invasion and migration abilities among the five cell lines. * P<0.05, ** P<0.01, *** P<0.001, vs control (Cas9) group.

We assessed the expression of proteins extracted from the five cell lines using western blot analysis. This procedure was repeated three times. The protein level was quantified by the grayscale ratio using ImageJ software, and statistical significance was determined using the GraphPad Prism 8 software (La Jolla, Fig 2A). Among the three steady knockout cell lines, the knockdown efficiency of pX459-AEG-1-3 was the highest (99%, vs. Cas9 group). AEG-1 protein expression in the five cell lines were 100%, 80%, 30%, 20.5%, and 1%, respectively, vs. the U251 cell line.

#### Flow cytometric analysis of apoptosis and the cell cycle

The effect of AEG-1 on the apoptosis of U251 cells was determined using flow cytometry (Fig 2B). The ratio of apoptosis among the five groups is shown in Fig 2D. PX459-AEG-1-3 had the highest early apoptosis and late apoptosis rates, especially the early apoptosis rate, which was 3.6-fold higher than that in the Cas 9 group. The cell cycle distribution results are shown in Fig 2C, and the percentage of cells in G2 increased in the three AEG-1 knockout groups (Fig 2E). Similarly, pX459-AEG-1-3 showed the highest ratio, and the percentage of cells blocked in the G2/M phase increased by approximately 62%.

#### Transwell invasion assays and scratch wound experiments

Cell migration was determined via scratch experiments. The cell migration rate was calculated as follows: relative mobility (%) = (1−48 h healing distance/0 h scratch distance) ×100%. The data are presented as mean ± SD. The results indicated that the invasion and migration abilities of the three experimental groups were reduced (Table 3, Fig 2F), particularly in the pX459-AEG-1-3 group.

**Table 3:** Related data of the Transwell invasion chamber experiments and scratch test.

Group	Number of invaded cells	Migration distance (l/μm)	Relative mobility (%)
U251	45.67±2.94	73.57±2.01	53.38±1.09
Cas9	42.3±2.16	44.10±2.37	36.99±1.83
pX459-AEG-1-1	12.00±1.41	21.4±2.63	18.38±2.05
pX459-AEG-1-2	4.67±0.82	10.36±1.54	9.29±1.28
pX459-AEG-1-3	0	5.60±1.83	5.11±1.35

#### ^1^H NMR spectroscopy

**Fig 3:**
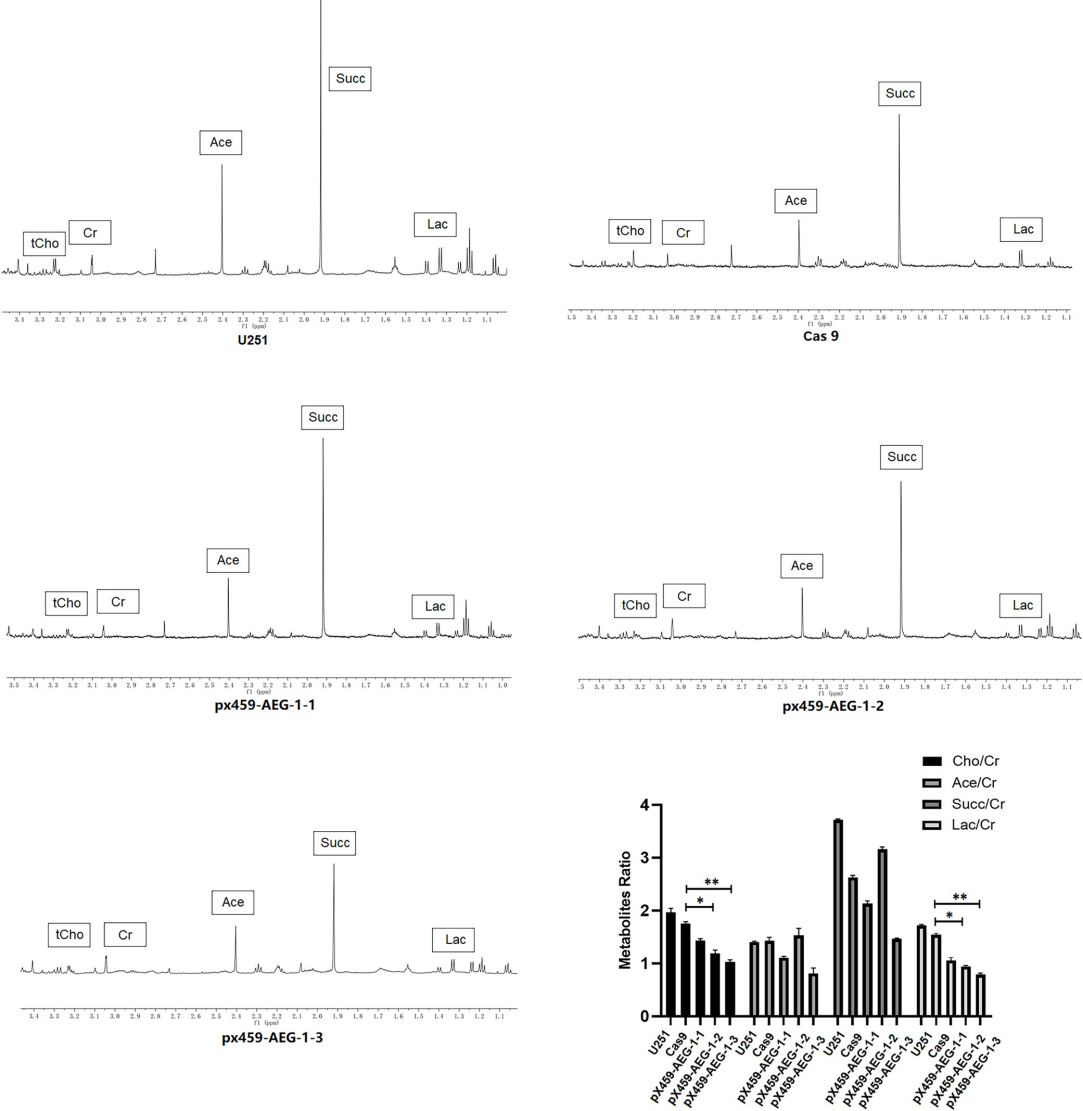
^1^H MRS spectra of the five cell lines. Metabolites labelled are as follows: Lac: lactate(1.33–1.34ppm), Succ: succinate(1.92ppm), Ace: acetate (2.40ppm),Cr: creatine(3.04ppm), tCho: total choline(3.21–3.23ppm).

^1^H MRS demonstrated content changes in total choline (tCho), creatine (Cr), acetate (Ace), succinate (Succ), and lactate (Lac) in these five cell lines. Distinct differences in relative metabolite concentrations were observed in the five cell lines (Fig 3). The spectra were phased, and the creatine peak at 3.04 ppm was used to reference the signals. We discovered that the tCho/ Cr ratio and Lac/ Cr ratio demonstrated a noticeable decline in the pX459-AEG-1-2 and pX459-AEG-1-3 groups and had the same downward trend as the AEG-1 protein expression, rate of apoptosis, and metastatic capacity of U251 cells. The tendency of the Ace/Cr ratio and the Succ/Cr ratio was not clear.

#### Correlation analysis

**Fig 4:**
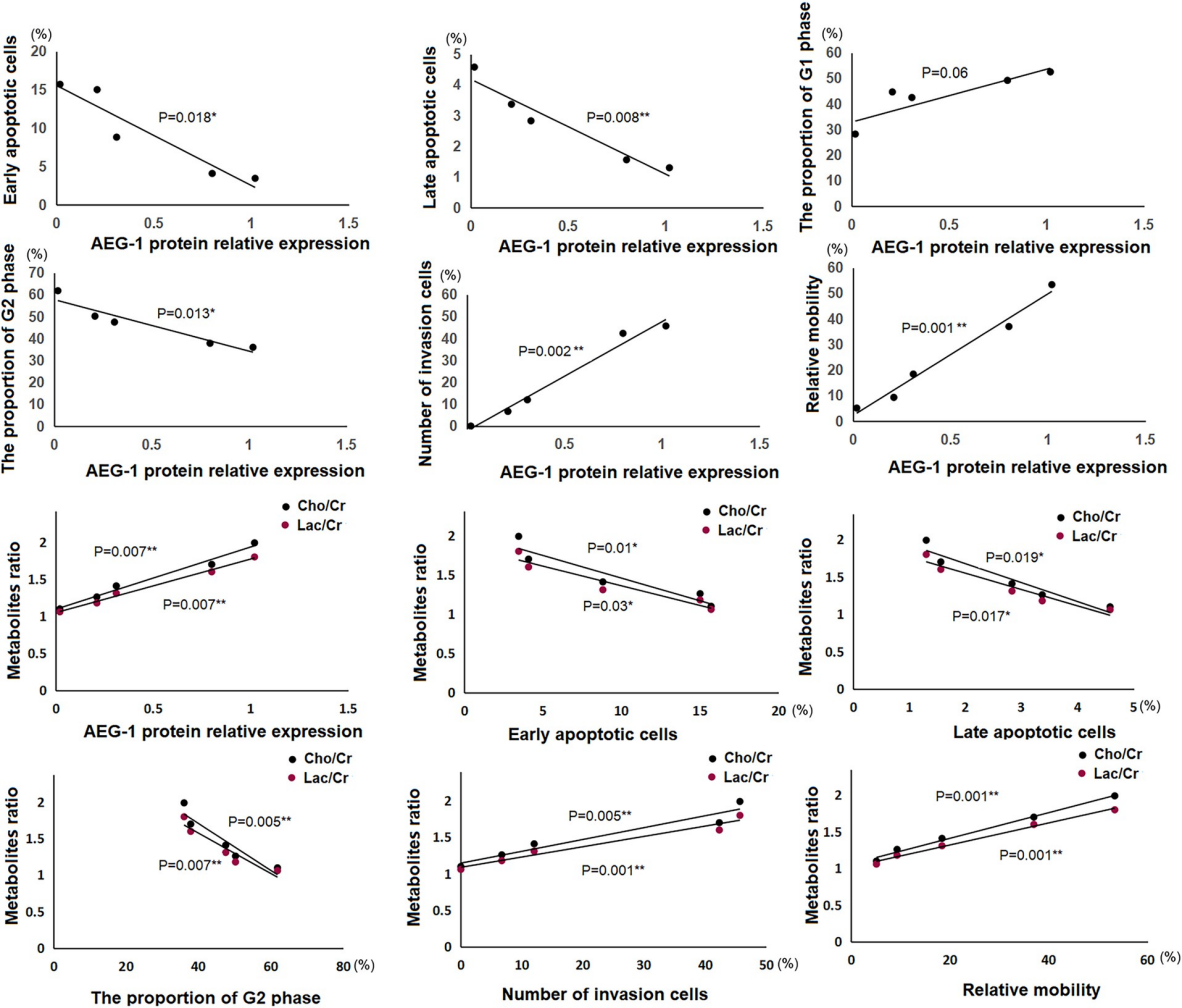
The correlation analysis among the biological behavior, AEG-1 protein relative expression level, and metabolite alterations in the U251 cell line.

In the five cell lines, several correlation lines based on the Pearson coefficient of correlation validated correlations among AEG-1 protein relative expression, biological behavior, and metabolite alterations (Fig 4).

## Discussion

Although glioma stem cells have the potential to proliferate and differentiate indefinitely and play a key role in the treatment, prognosis, and recurrence of glioma [36], the process of culturing and purifying glioma stem cells is complex, whereas U251 cells can embody most of the properties of glioma. Previous researchers have reported that the CRISPR/Cas system is effective in the gene editing of U251 cells. Therefore, we used U251 cells as the research object instead of glioma stem cells in the present investigation. The CRISPR/Cas9 system comprises a single guide RNA (sgRNA) and the DNA endonuclease Cas9, with the former directing the latter to specific DNA sequences to cut double-stranded DNA site-specifically. Different sgRNAs guide the knockdown of target genes at various sites, resulting in multiple mutations and relevant knockdown efficiencies [37]. This study used the CRISPR/Cas9 system to knock down the AEG-1 gene in U251 cells. We designed three sgRNA sequences (Table 1) targeting AEG-1 and found that the gene mutation forms(Fig 1E) and knockout efficiency of the three sgRNA sequences varied (Fig 2A). Therefore, we constructed three monoclonal AEG-1-knockout U251 cell lines and then evaluated their biological behavior and metabolite ratio. The five cell lines include the U251 cell line, the Cas 9 cell line (transferred into the empty Cas 9 carrier without sgRNA), and three AEG-1-knockout cell lines (pX459-AEG-1-1, pX459-AEG-2-2, pX459-AEG-3-3). Among the three steady knockout cell lines, the knockdown efficiency of pX459-AEG-1-3 was the highest, this is because the AEG-1-sgRNA-3 primer and the AEG-1 gene have a better fit and can assist in maximizing the knockdown of the AEG-1 gene during the guided knockdown process.

Our study exhibited that AEG-1 knockout leads to cell cycle arrest in the G2 phase (Fig 2C and 2E), resulting in increased levels of apoptosis (Fig 2B and 2D). The G2 phase proportion negatively correlated with the AEG-1 expression, and the ratio of apoptosis had a negative correlation with the AEG-1 protein relative expression (Fig 4). The expression of AEG-1 is regulated by the Ha-ras gene and PI3K/Akt-GSK3β-c-Myc signaling pathway in various malignancies [38–41]. Upregulation of AEG-1 expression further activates the PI3K/Akt signaling pathway, and AKT acts downstream of AEG-1, where activation of AKT leads to GSK3B phosphorylation and repression of FOXO1/3 phosphorylation [42, 43]. The AEG-1 and Ha-ras genes cooperate to regulate the expression of transcription factors, such as FOXO1, Miz-1, P53, AP-1, and other downstream genes of the PI3K/Akt signaling pathway, thereby mediating the abnormal proliferation and anti-apoptotic ability of tumor cells [43–48]. Previous studies have indicated the influence of AEG-1 upregulated expression on downstream signal factors of Akt signal routing connected with cell proliferation and growth. AEG-1 is also a downstream target molecule of the c-myc gene [47, 49, 50], a major cell cycle checkpoint regulatory factor. Consequently, the downregulation of AEG-1 should reduce proliferative capacity, induce cell cycle arrest, and promote apoptosis in U251 cells.

Based on the experimental results of the cell biological behavior of the five cell lines (Fig 2F), our study demonstrated that knockout of AEG-1 could significantly inhibit the proliferation, migration, and invasion of the U251 cell line, and cell metastasis capacity had positive correlations with AEG-1 relative expression (Fig 4). AEG-1 is considered a significant stimulatory regulator of NF-κB, and activation of NF-κB via AEG-1 represents a key signaling pathway through which AEG-1 promotes independent anchored growth and tumor progression in malignant glioma cells [39, 44, 51]. AEG-1 may act as a linker between p65, NF-κB, and cAMP response element binding protein (CBP), promoting transcriptional upregulation of genes downstream of NF-κB, which is essential for tumor metastasis and invasion [52–54]. Therefore, the invasive metastatic ability of U251 cells was reduced after the down-regulation of the AEG-1 gene, as shown in the experimental results.

Our study also explored the relationship between metabolite alterations (Fig 3), oncogene AEG-1 expression, and biological behaviors in U251 cells. The tCho/Cr ratio and Lac/Cr ratio positively correlated with the AEG-1 expression and invasion and migration abilities, but had passive correlations with the ratios of apoptosis and G2 phase proportion (Fig 4). Metabolic abnormalities are a major hallmark of cancer [55]. Aberrant tumor metabolisms, such as aerobic glycolysis and increased anabolic pathways, are important in tumorigenesis, metastasis, drug resistance, and tumor stem cells [56, 57]. AEG-1 is an active element in tumorigenesis and progression, and AEG-1 promotes the production of cell membranes [4]. Choline-containing compounds are pivotal parts of cell membranes, playing crucial roles in membrane turnover, are correlated with cell density, and are therefore used to mirror abnormal cellular proliferation [58]. In several in vivo MRS clinical studies, choline assessment was a potential indicator of malignant glioma [59, 60]. Theoretically, the total choline content should be relevant to AEG-1 expression, consistent with our experimental results. The Warburg effect reveals that malignant tumors are metabolic diseases, with many of them generating ATP through glycolysis during cell growth [12]. The 5-adenosine monophosphate (AMP)-activated protein kinase (AMPK) promotes anabolic processes and the Warburg effect in cancer by inducing multiple glycolytic enzymes [61]. In glioblastoma cells, AEG-1 acts as a glycolytic regulator, sensing the energy status of the cell, lowering the ATP/AMP ratio, and activating AMPK and its downstream targets, thereby maintaining sufficient amounts of ATP required for tumor proliferation and enhancing the glycolytic flux of the cell [62]. Numerous scholars have experimented with exerting glycolysis metabolite concentration to supplement the supervision of glioma therapy [29, 63, 64]. Glycolysis offers energy for tumor cells and generates lactate, and variation in lactate content may reflect the AEG-1 gene expression in glioma cells, which is consistent with our experimental results. Therefore, the tCho/Cr and Lac/Cr ratios could be recommended as non-invasive imaging biomarkers reflecting the AEG-1 expression in glioma cells.

Gliomas are among the most prevalent and aggressive tumors of the central nervous system, with a poor prognosis and low survival rates. Overexpression of AEG-1 has been found in glioma tissues, and its expression correlate with the clinicopathological classification of gliomas. Thus, AEG-1 may be a potential target for glioma therapy. Understanding the ^1^H MRS alterations that occur at different AEG-1 oncogene expression in U251 cells in vitro should facilitate the screening of glioma-associated biomarkers. Under certain circumstances, the results of these in vitro experiments can be processed and translated into relevant in vivo studies to aid accurate clinical diagnosis and treatment, providing the basis for future targeted molecular therapies. Therefore, our study, using metabolite changes to monitor the AG-1 expression and its biological behavior in U251 cells, is significant.

The new paragraph is used to report the limitations of the study as follows:

The report has the following limitations. Glioma stem-like cells are often the subject of research exploring new therapeutic approaches, and the use of the stable U251 glioma cell line for this study was inappropriate. An additional deficiency of this study was the expression of the associated molecular pathways (PI3K/Akt-GSK3β-c-Myc, PP53, NF-κB, AMPK, etc.) was not detected simultaneously. Also, the effect of iron death on apoptosis in glioma cells as well as the effect of oxidative phosphorylation on the Warburg effect were not considered in this research. Then, due to the restriction of experimental conditions, we did not obtain enough NMR spectral information to fully demonstrate the correlation between metabolism and AEG-1 knockdown. All of these limitations will continue to be improved in future studies.

## Conclusions

In summary, this study demonstrated the feasibility of direct gene modification of the oncogene AEG-1 in glioma cells using the CRISPR/Cas9 system. Simultaneously, our MRS-detected choline and lactate metabolites may have the potential as non-invasive biomarkers for monitoring AEG-1 expression levels to assist clinical work.

## Supporting information

S1 File (PDF)

S1 Raw images (TIF)

S2 Raw images (TIF)
