# Supplementary figures and images for: Using the metabolite alterations monitoring the AEG-1 expression level and cell biological behaviour of U251 cell in vitro
Source: PLoS One. 2023 Sep 1;18(9):e0291092. doi: 10.1371/journal.pone.0291092 (PMC10473485; doi:10.1371/journal.pone.0291092)

1.Original drawing of Fig 1C.

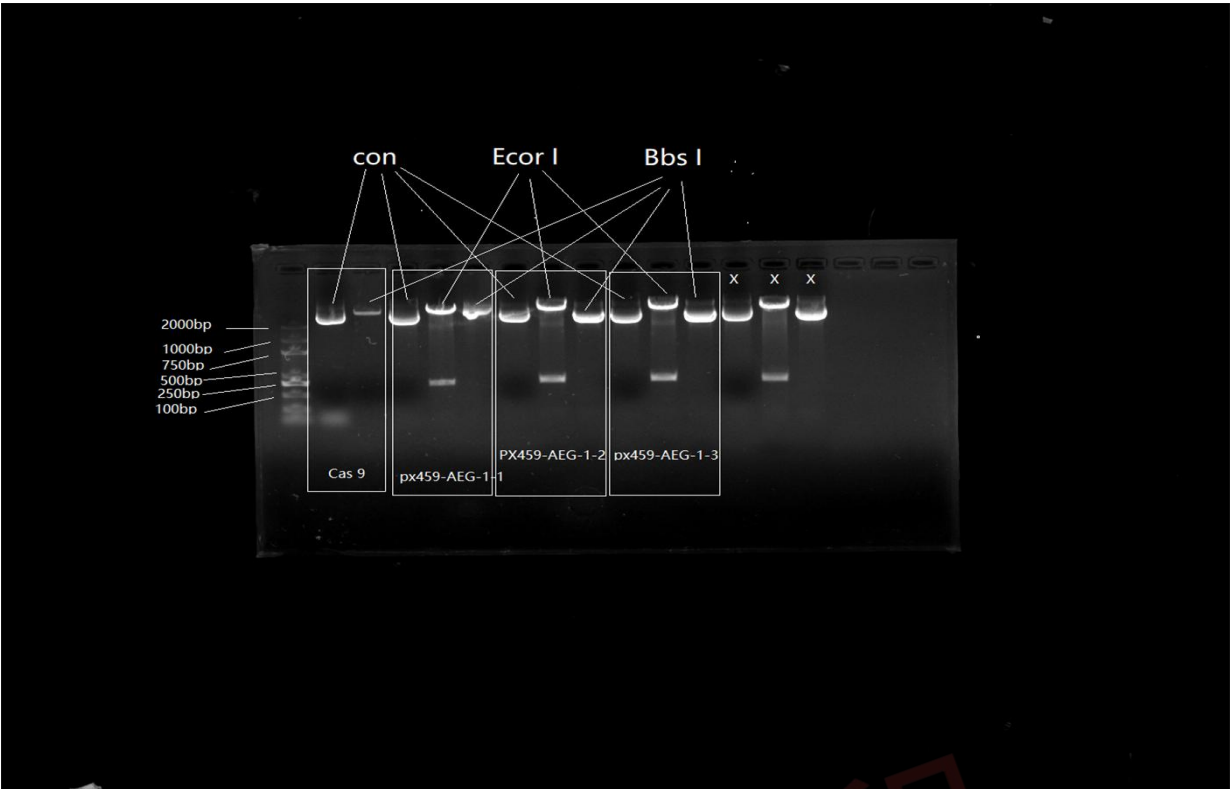

2.Original drawing of Fig 2A

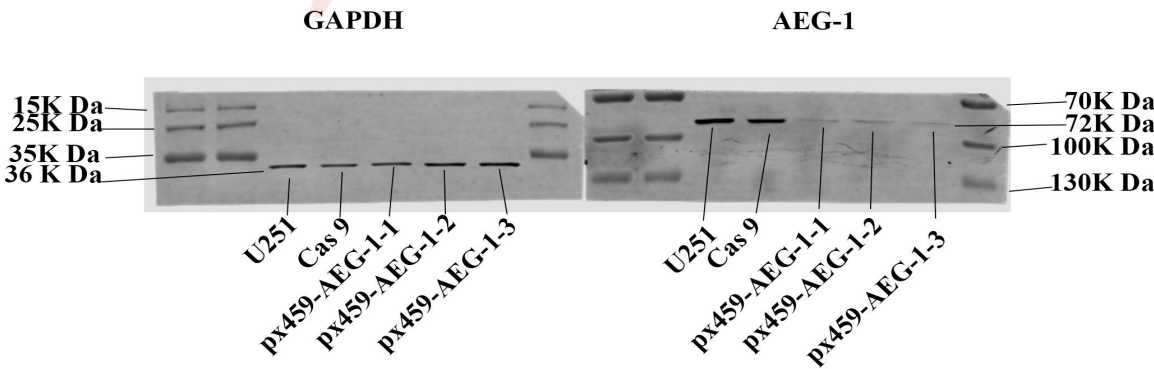

Supplement: S1 File — (PDF) [file pone.0291092.s001.pdf]

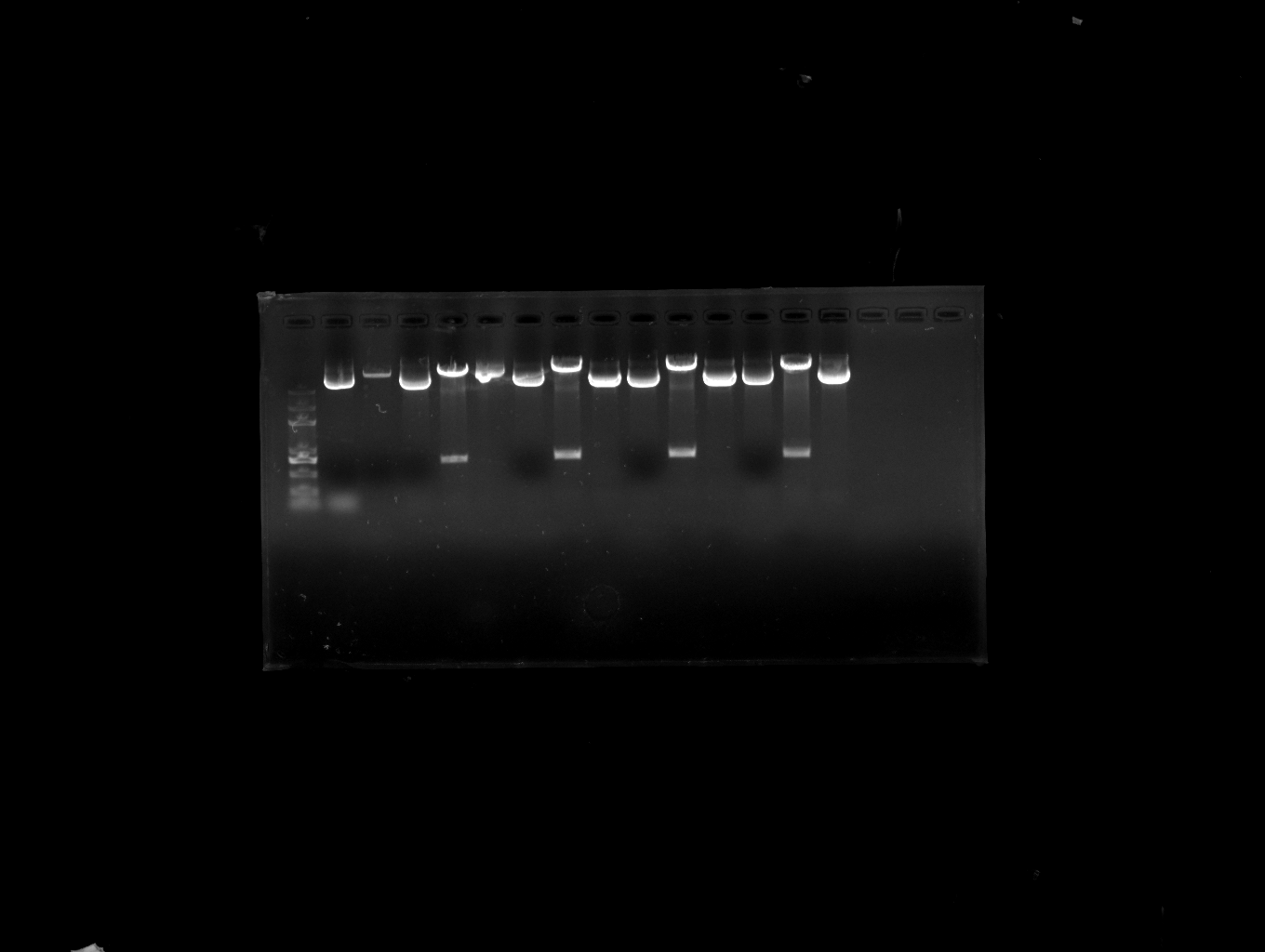

Supplement: S1 Raw images — (TIF) [file pone.0291092.s002.tif]

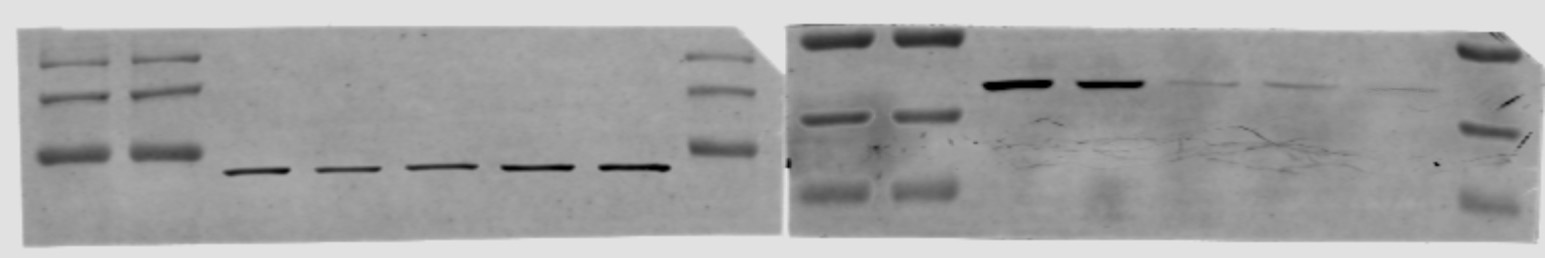

Supplement: S2 Raw images — (TIF) [file pone.0291092.s003.tif]
